# miR-671-5p Blocks The Progression Of Human Esophageal Squamous Cell Carcinoma By Suppressing FGFR2

**DOI:** 10.7150/ijbs.32429

**Published:** 2019-07-21

**Authors:** Xiaoyan Li, Changjun Nie, Baoqing Tian, Xuan Tan, Wei Han, Jiakang Wang, Yuan Jin, Yadan Li, Xinyuan Guan, An Hong, Xiaojia Chen

**Affiliations:** 1 Institute of Biomedicine & Department of cell Biology, Jinan University, Guangzhou, Guangdong, 510632, P. R. China.; 2 National Engineering Research Center of Genetic Medicine, Guangzhou, Guangdong, 510632, P. R. China.; 3 Guangdong Provincial Key Laboratory of Bioengineering Medicine, Guangzhou, Guangdong, 510632, P. R. China.; 4 Cancer Center of Guangzhou Medical University, Guangzhou, Guangdong, 510090, P. R. China.; 5 Department of Clinical Oncology, University of Hong Kong, Hong Kong, China

**Keywords:** FGFR2, miR-671-5p, ESCC, tumor progression

## Abstract

Esophageal cancer is the eighth most common malignant tumor worldwide, of which esophageal squamous cell carcinoma (ESCC) is the dominant histological subtype. A drug shortage for ESCC therapy triggered us to explore the roles of fibroblast growth factor receptor 2 (FGFR2) and its upstream regulator miR-671-5p in ESCC progression. We compared the levels of FGFR2 and miR-671-5p between human ESCC tissues and their matched normal esophageal tissues and found an association between higher levels of FGFR2 and lower levels of miR-671-5p in ESCC tissues. High levels of FGFR2 resulted in the activation of the ERK and AKT pathways and a promotion of ESCC progression. High levels of miR-671-5p specifically reduced the expression of FGFR2 and suppressed ESCC progression in both in vitro *a*nd in vivo models. Therefore, suppressing FGFR2 and enhancing miR-671-5p expression may be the right approaches for ESCC therapy.

## Introduction

Esophageal cancer is the eighth most frequent and the sixth most fatal cancer type worldwide, and is highly prevalent distributed in Asia and Africa 1. According to its tissue origin, esophageal cancer is divided into esophageal adenocarcinoma and esophageal squamous cell carcinoma (ESCC), the dominant histological subtype 2. The overall 5-year survival rate for patients with ESCC at advanced stages is only approximately 10-20% 3. Therefore, understanding the mechanism of ESCC progression is very important for future therapy.

Fibroblast growth factor receptors (FGFRs) belong to a family of receptor tyrosine kinases, comprising of five members, FGFR1 to FGFR5, and are critical for not only normal development but also tumorigenesis 4-7. FGFRs bind to their extracellular ligands fibroblast growth factors, which induces receptor dimerization in the cell membranes, leading to trans-auto-phosphorylation of the intracellular tyrosine residues. This phosphorylation results in the activation of the downstream signaling pathways, such as MAPK, PI3K-AKT and PLCγ, and plays key roles in cellular anti-apoptosis, angiogenesis, proliferation, and migration 8, 9. Mutations or abnormal expression of FGFRs may result in constitutive dimerization of receptors and auto-activation of kinases, thereby inducing aberrant intracellular signaling 8. FGFR2 is generally considered as an oncogene. For example, overexpression of FGFR2 promotes the proliferation and survival of gastric cancer cells through activating the downstream MAPK/ERK and PI3K/AKT signaling pathways 10, and FGFR2 enhances the phosphorylation activity of RSK2 and regulates the migration of human mammary epithelial cells 11. Previous evidences have shown that FGFR2 is a potential therapeutic target, but the role of FGFR2 in ESCC progression has rarely been reported.

miRNA is a kind of small non-coding RNA, approximately 20-30 nucleotides in length. It inhibits translation or promotes the degradation of targeting mRNA by binding to its 3′-untranslated region (3′-UTR) to regulate gene expression 12-15. It has been reported that several miRNAs activate or inhibit tumor progression 16. For example, miR-3188 significantly inhibits the development of nasopharyngeal carcinoma by targeting mTOR to inactive the p-PI3K/p-AKT/p-mTOR pathway 17; and the deficiency of miRNA-150 leads to inhibition of differentiation of intraepithelial lymphocytes 14. miR-671-5p mentioned in this study, promotes the migration and proliferation of human glioblastoma multiforme by targeting CDR1-AS, CDR1, and VSNL1 18, and suppresses the development of breast cancer via inhibiting epithelial-to-mesenchymal transition by targeting FOXM1 19. High levels of miR-671-5p and miR-193a-5p inhibit oncogene *SMARCB1/INI1* expression in pediatric chordomas 20. miR-206, miR-381, and miR-671-5p suppress the expression of *SMARCB1* in epithelioid sarcoma 21. However, the roles of miR-671-5p in ESCC progression are still unclear.

Here, we showed that suppressing the expression of FGFR2 led to significant decreases in ESCC xenograft size. Most of the human ESCC tissues displayed higher levels of FGFR2 and lower levels of miR-671-5p compared with their matched normal esophageal tissues. Furthermore, miR-671-5p targeting the FGFR2 3′-UTR was used to reduce the expression of FGFR2, which suppressed the proliferation, migration, invasion, and xenograft growth of ESCC cells.

## Materials and Methods

### Collection of normal esophageal and ESCC tissues and culture of human ESCC cell lines

Thirty-five pairs of ESCC tumor tissues and their matched normal esophageal tissues were collected for immunochemical analyses of FGFR2 protein as previously described 22, and six pairs of ESCC tumor tissues and their matched normal esophageal tissues were collected for analyses of miR-671-5p. All tissues were provided by the Cancer Center of Guangzhou Medical University, Guangzhou, China. None of the patients received prior chemo-radiotherapy. The relative intensities of FGFR2 in the tissue sections were unbiasedly estimated by researchers based on a scale from 0-10. A line of normal esophageal cells and eight lines of ESCC cells were maintained in RPMI medium (HyClone, USA) supplemented with 10% fetal bovine serum (FBS) at 37 °C in an incubator with a humidified atmosphere of 5% CO_2_. All procedures performed in studies involving human patients were in accordance with the ethical standards of the Institutional and/or National Research Committee and the Declaration of Helsinki and its later amendments, or comparable ethical standards. This study was in accordance with the ethics review regulations, and was reviewed and approved by the Human Subject Research Ethics Committee of Cancer Center of Guangzhou Medical University.

### RNA extraction and quantification by quantitative real-time PCR

Total RNA was extracted from cells using RNAiso Plus, according to the manufacturer's protocol (Takara). The cDNA was synthesized using a reverse transcription kit (Takara). qPCR (Real-time Quantitative PCR Detecting System) was performed using CFX96 Touch^TM^ Real Time PCR Detection System with SYBR Green Dye mix (Takara). The relative expression levels of mRNA or miRNAs were calculated by the comparative C_T_ method and normalized to the levels of GAPDH or U6, respectively. The primer sequences are shown in Table 1.

### Creation of stable cell lines using lentivirus system

We used ta lentivirus system to create stable cell lines with overexpression or knocked-down expression of FGFR2. The vector pCDH-FGFR2 was created by ligating the DNA fragment of the FGFR2 gene with the vector pCDH-CMV-MCS-EF1-GreenPuro (Cat. #CD513B, SBI, CA), digested by* XhoI1* and *BamH1*, and co-transfected into 293T cells with the packaging vectors psPAX2 and pMD2G, using Lipofectamine 3000 reagent (Invitrogen, CA) according to the manufacturer's protocol. Stable cell lines were selected based on their resistance to puromycin. Following the same procedure, both shFGFR2-1 (shRNA-1, TRCN0000231053, BROAD) and shFGFR2-4 (shRNA-4, TCRN0000218493, BROAD) oligonucleotides were designed and cloned into the pLKO vector to create their respective stable cell lines.

### Assays for cell proliferation and colony formation

ESCC cells (1.5×10^3^ cells per well) were seeded in 96-well plates, and cultured for 1-7 days to achieve lentivirus-mediated overexpression or knock-down FGFR2, and for 1-4 days to test the impact of transient transfection with miR-671-5p mimics or miR-671-5p inhibitor, respectively. Subsequently, the rates of cells proliferation were measured using the cell Counting Kit-8 (CCK8) kit according to the manufacturer's protocol. For colonies formation assays, ESCC cells (1000 cells per well) were seeded in 6-well plates, and cultured for 7 days at 37 °C in an incubator with 5% CO_2_. The number of colonies formed in each well was counted after the cells in each well were fixed with 4% paraformaldehyde for 10 min, and then stained with crystal violet for 10 min.

### Assays for cell migration and invasion

Assays for cell migration and invasion were performed using Transwell inserts (Costar, USA) and invasion using Matrigel-coated plates (BD Bioscience, USA), respectively. Approximately, 5×10^4^ tumor cells in serum-free medium were placed in the upper chamber, and cells in medium with 20% FBS were placed in the lower chamber. Cells were incubated for 36h, fixed with 4% paraformaldehyde, and stained with crystal violet. The number of cells that attached on the underside of the filter was counted under a microscope.

### Animal experiments

All experimental procedures were performed according to the institutional ethical guidelines approved by the Institutional Animal Care and Use Committee of Jinan University. Male 4-5 week-old BALB/c nude mice were subcutaneously injected with 3×10^6^ KYSE180-CTL, KYSE180-shFGFR2-4, EC109-CTL, or EC109-FGFR2 cells to establish their respective ESCC xenografts. To test the impact of miR-671-5p, the same mice were subcutaneously injected with 3×10^6^ KYSE180 cells to establish ESCC xenografts with a size of 1 cm in diameter, and then 1 nM miR-671-5p mimics and its negative control were injected into the intra-tumor spaces. Tumor growth in mice was monitored on daily base and tumor volume was calculated with the following equation: *V=0.5xLxW^2^* 23.

### Immunoblot analysis

Immunoblot analysis was performed as previously described 24, 25. The following antibodies were used: anti- FGFR2, from Santa Cruz Biotechnology, anti-GAPDH from Sigma-Aldrich, and anti-AKT, anti-phosphor-AKT, anti-ERK, anti-phosphor-ERK, anti-cyclinD1, anti-cyclinB1, and anti-phosphor-FGFR from Cell Signaling Technology. Secondary antibodies were from Sigma-Aldrich. Densitometry (Bio-Rad Laboratories) was applied to analyze the intensities of the respective protein bands.

### Prediction of miR-671-5p target genes and dual luciferase reporter assay

TargetScan, miRwalk, miRanda and miRDB softwares were used to predict the target genes of miR-671-5p and FGFR2-3′-UTR was identified as the target sequence. The 3′-UTR of human FGFR2 mRNA fragment was amplified by PCR using the listed primers (Table 1) and cloned into psi-CHECK-2 vector to create WT (Wild-type) vector. Site-Directed Mutagenesis of miR-671-5p binding site in FGFR2-3′-UTR (Mutation type, MT vector) was created using Gene Tailor Site-Directed Mutagenesis System (Invitrogen). For luciferase reporter assays, cells were transfected with WT or MT vector and treated with miR-671-5p mimics or its inhibitor for 48 hours. Luciferase activities were measured using the Dual-Luciferase Reporter kit (Promega, USA) and normalized to the activities of firefly luciferase.

### miRNA transfection of Cells

miR-671-5p mimics (miR-671-5p) and its negative control (mimics Nc), and miR-671-5p inhibitor and its negative control (inhibitor Nc) were designed and synthesized by RiboBio (Guangzhou, China). miR-671-5p mimics and mimics Nc were transfected into KYSE180 cells and miR-671-5p inhibitor and inhibitor Nc were transfected into EC109 cells using Lipofectamine 3000 transfection reagent (Invitrogen) according to the manufacturer's protocol.

### Statistical analysis

Statistical analysis was performed using SPSS 19.0 statistical software. Data were expressed as means ± standard deviation (SD). The statistical significances of differences between two independent groups was tested by Student's* t*-test and expressed as *, *p < 0.05*; **, *p < 0.01*; and ***, *p < 0.001*.

## Results

### Levels of FGFR2 are elevated in ESCC tissues and cell lines

**Figure 1:**
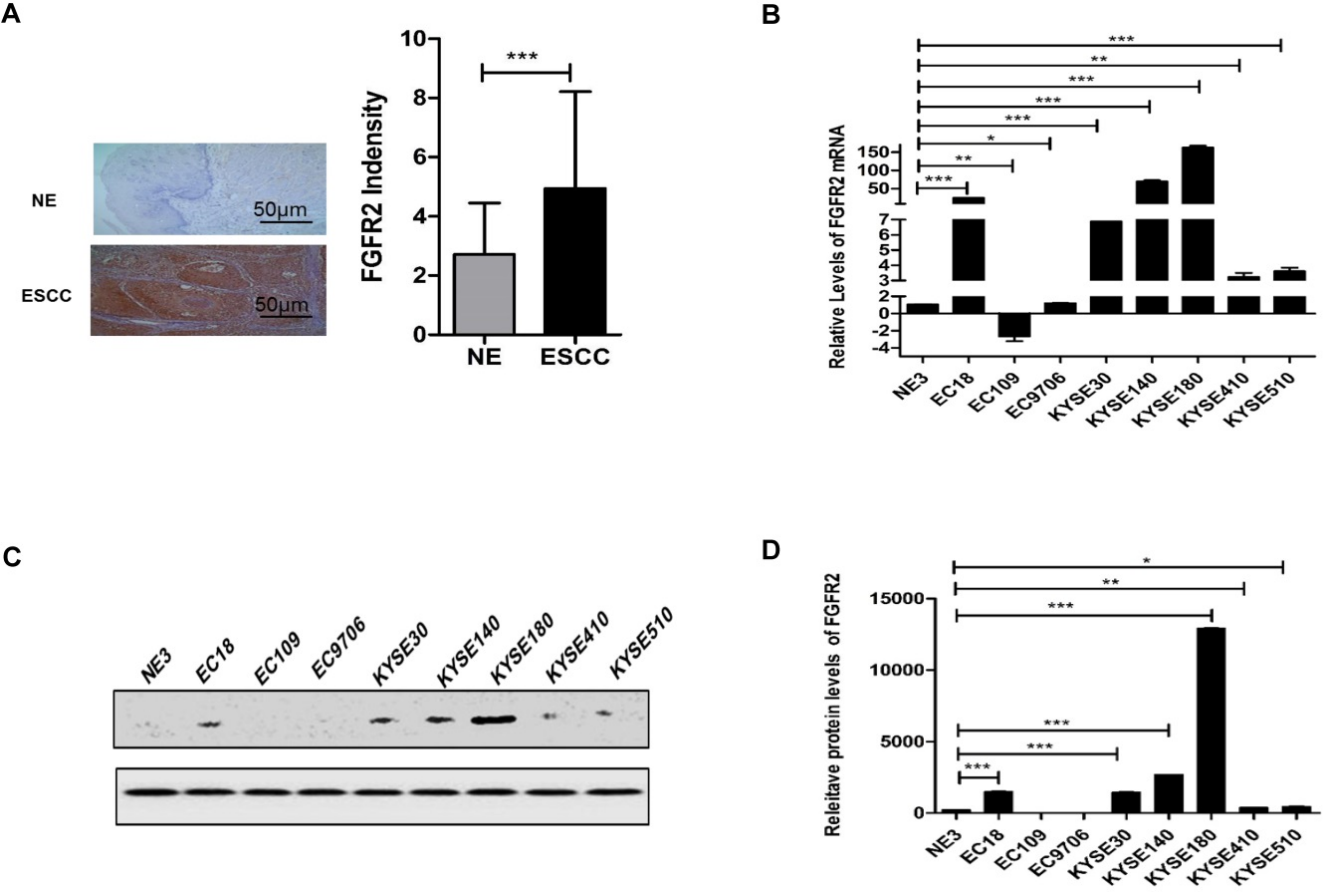
** Expression of FGFR2 in normal and ESCC tissues and cells. A.** Representative images and a quantification analysis showing the levels of FGFR2 in 35 pairs of normal esophageal tissues (NE) and esophageal squamous cell carcinoma (ESCC) tissues as detected by immunohistochemical staining. Bar=50μm. **B.** A plot showing the relative levels of FGFR2 mRNA in normal esophageal cell line NE3 and several ESCC cell lines. Data shown are the means and standard deviations (Mean±S.D.) of at least three repeats. The significances of difference between ESCC cell lines and NE3 are analyzed using Student's T-test. Here and later, * indicates *p* < 0.05; ** *p* < 0.01; and *** *p* < 0.001.**C,D** Western blot showed the FGFR2 expression levels in NE3 and several ESCC Cell lines.

Previously, we reported that the expression levels of FGFRs between carcinoma and para-carcinoma cells in patients with digestive or reproductive system cancers are significantly different, and FGFR2 and FGFR4 are closely related to the susceptibility of digestive and reproductive system cancers 22. We further evaluated the expression levels of FGFR2 in normal and ESCC tissues from 35 pairs of human patients by immunohistochemistry. We found that the levels of FGFR2 in ESCC tissues were significantly higher than those in normal tissues (Fig. 1A) although there was no significant difference between different groups with different clinical parameters (Table 2). We also examined the levels of FGFR2 mRNA in a panel of ESCC cell lines by qPCR assays, and found that majority of the ESCC cell lines, except EC109 had significantly higher levels of FGFR2 mRNA than the immortalized NE3 cell line derived from normal human esophageal tissues (Fig. 1B-D). Therefore, our results suggested that ESCC tissues and cell lines express higher levels of FGFR2.

### FGFR2 promotes proliferation, colony formation, migration, invasion, and tumorigenesis of ESCC cells

**Figure 2:**
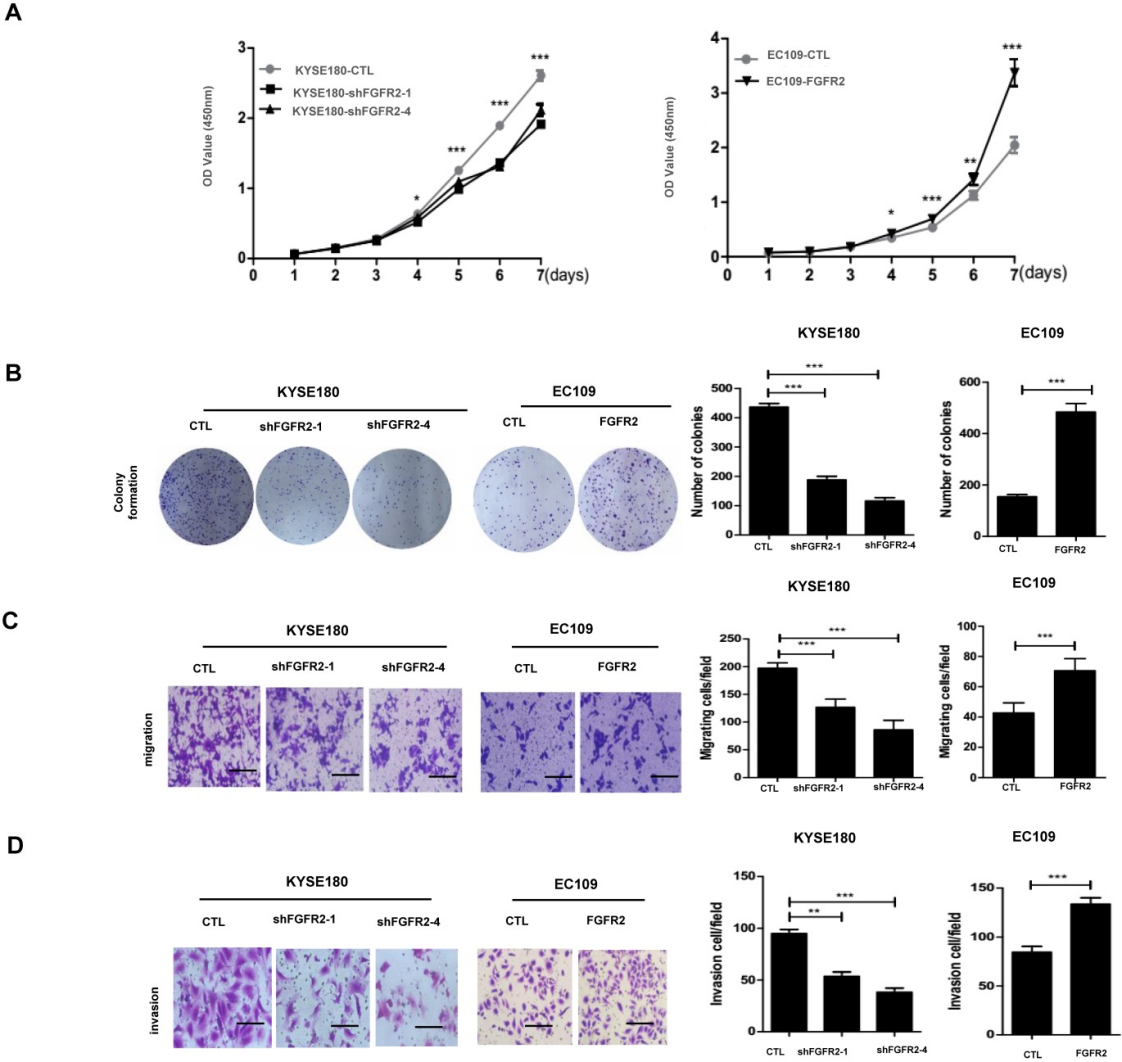
** FGFR2 promotes ESCC progression *in vitro*. A.** Plots showing the relative densities of KYSE180 cells treated with random shRNA (CTL), shFGFR2-1 or shFGFR2-4, and EC109 transfected with control plasmid (CTL) or plasmid expressing FGFR2. **B-D.** Representative images and plots showing the relative levels of colony formation (**B**), migration (**C**) and invasion (**D**) in the same cell lines as described in panel **A**. Bar=100μm.

**Figure 3:**
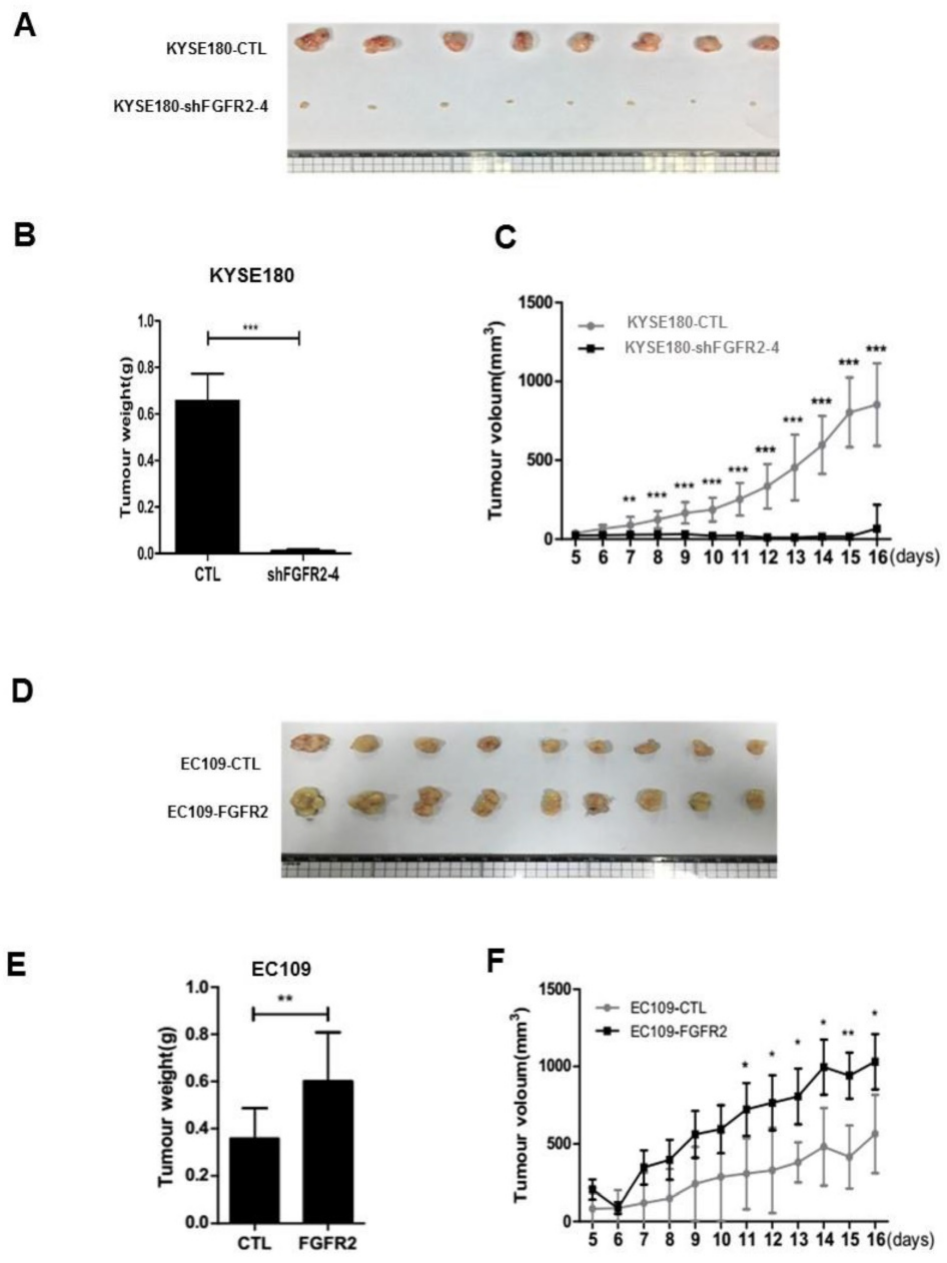
** FGFR2 promotes the ESCC progression *in vivo*. A-F.** Images (**A,D**) and plots of weights (**B,E**) and volumes (**C,F**) of tumors formed in BALB/c nude mice injected of KYSE180 cells treated with random shRNA (CTL), shFGFR2-1 or shFGFR2-4 (**A-C**), or EC109 transfected with control plasmid (CTL) or plasmid expressing FGFR2 (**D-F**).

We selected KYSE180 cells with the highest levels of FGFR2 mRNA and EC109 cells with the lowest levels of FGFR2 to manipulate the expression levels of FGFR2. KYSE180 and EC109 cells were transfected with two types of shFGFR2 to silence and increase the expression of FGFR2, respectively. Two KYSE180 cell lines transfected with shFGFR2 displayed a reduction, while EC109 cells overexpressing FGFR2 displayed an increase in the rates of cell proliferation, colony formation, migration, and invasion compared with their respective controls (Fig. 2). We further investigated the effect of FGFR2 on ESCC progression *in vivo* using a xenograft tumor nude mouse model. Results showed that KYSE180 cells infected with shFGFR2 developed dramatically smaller tumors, while EC109 cells overexpressing FGFR2 developed significantly larger tumors as compared with their respective controls (Fig. 3). Therefore, our findings indicate that FGFR2 may act as an oncogene to promote ESCC progression*.*

### FGFR2 activates the ERK and AKT signaling pathways and alters cell cycle

**Figure 4:**
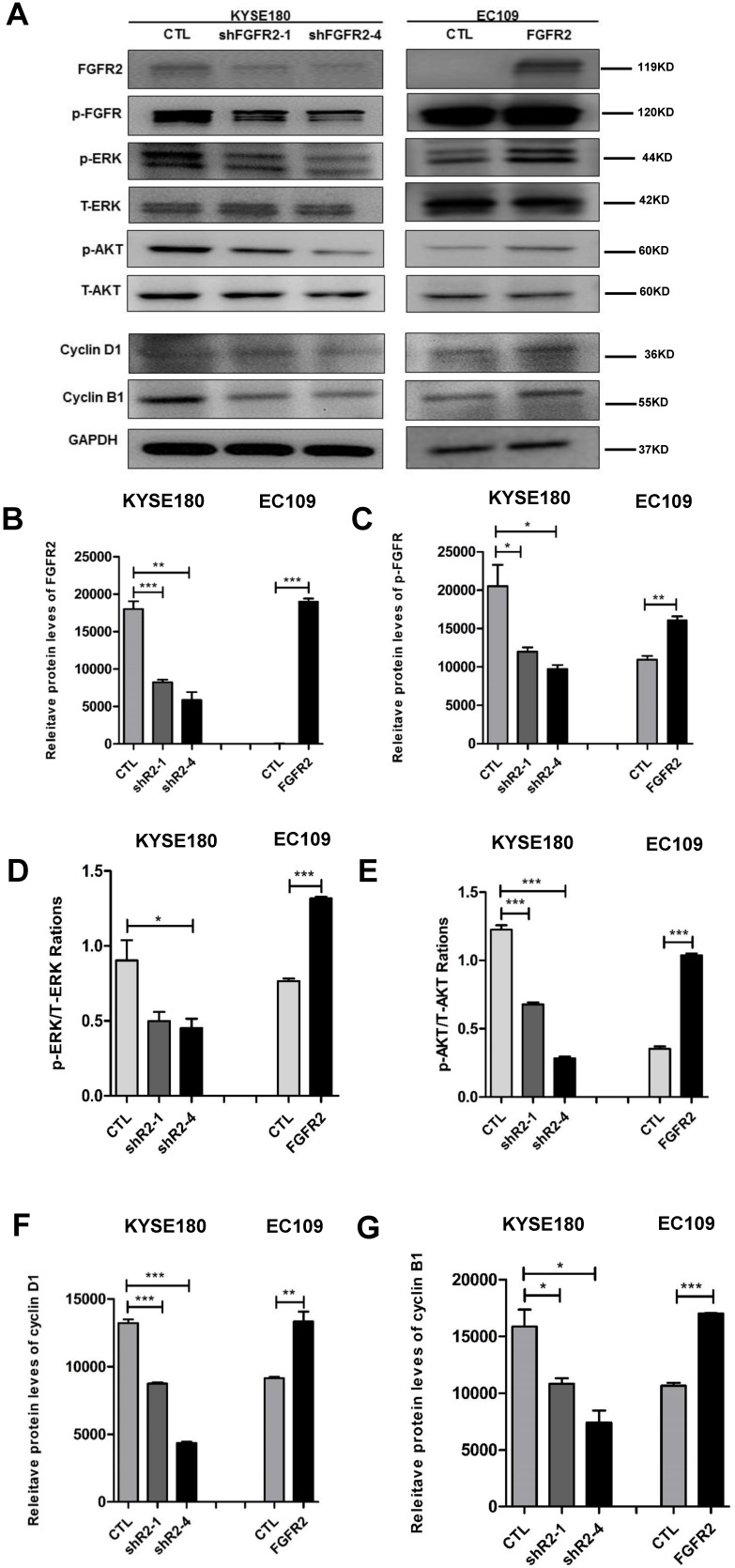
** FGFR2 activates the MAPK-ERK and PI3K-AKT pathways. A-E.** Representative immunoblots (**A**) and plots of relative levels of FGFR2 (**B**) and p-FGFR proteins (**C**), ratios of p-ERK to ERK (**D**), ratios of p-AKT to AKT (**E**), relative levels of cyclin D1 (**F**) and cyclin B1 proteins (**G**). GAPDH serves as loading control.

As expected, all cell lines expressed the expected levels of FGFR2 proteins (Fig. 4 A, B). The intensities of FGFR2 signals represented by the levels of phosphorylated FGFR were correspondingly altered (Fig. 4A, C). It has been reported that FGFR2 may impact tumorigenesis through ERK and AKT 26, 27. ERK is a classical signaling molecule involved in the MAPK signaling pathway, which is well-known to enhance cell differentiation, proliferation and motility 28, 29, while AKT is a key molecule connecting many pathways to influence cell survivals 30. Here, suppression of FGFR2 led to a decrease in the levels of the phosphorylated ERK (Fig. 4A, D) or AKT (Fig. 4A, E), while overexpression of FGFR2 led to an enhancement of these levels. In addition, cyclin D1 expression has been frequently reported to be elevated in a variety of tumors and may contribute to tumorigenesis 31; while cyclin B1 is a regulatory protein involved in cell mitosis 32. The levels of cyclin D1 and cyclin B1 were reduced in cells lines expressing lower levels of FGFR2 and increased in cells overexpressing FGFR2 (Fig. 4A,F,G). Therefore, FGFR2 may enhance cell proliferation through accelerating the cell cycle.

### miR-671-5p suppresses the levels of FGFR2 protein in ESCC tissues

**Figure 5:**
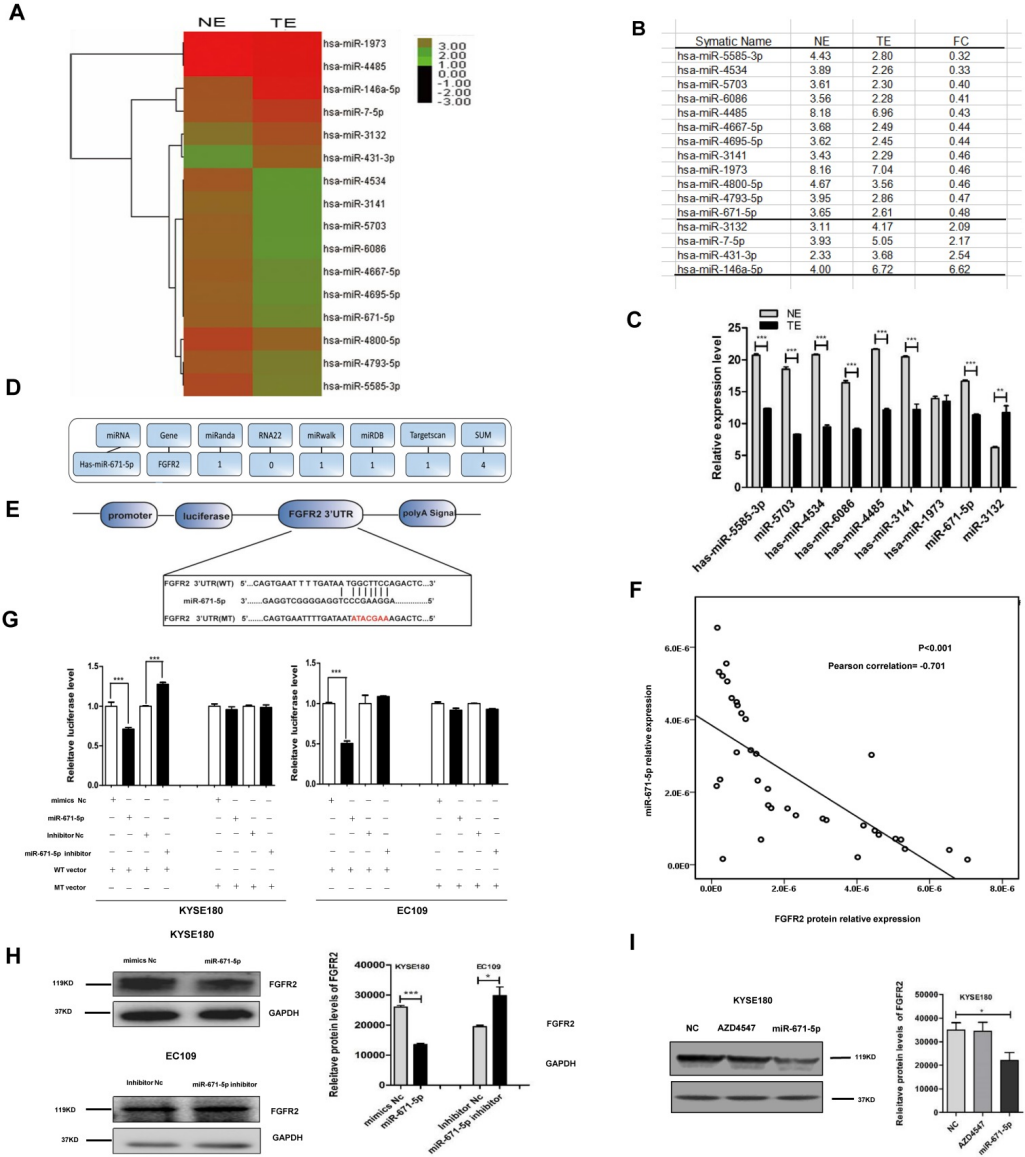
** miR-671-5p suppresses the expression of FGFR2 in ESCC. A,B**. A plot (**A**) and a table (**B**) showing the relative levels of miRNAs in normal esophageal squamous tissue (NE) and ESCC tumor tissue (TE) as detected with miRNA microarrays. FC, fold of change. **C.** Plots of relative expression levels of miRNAs as verified by qPCR. U6 serves as internal control. **D.** A diagram showing the procedure to use different Bioinformatics websites to predict whether miR-671-5p targets to FGFR2-3'-UTR. **E.** A diagram showing the binding site and sequence of miR-671-5p targeting to FGFR2-3'UTR and the sequences inserted in wild-type (WT) and mutant (MT) reporter vectors. **F.** A bivariate correlation analysis of the level of FGFR2 and miR-671-5p. **G.** Plots showing the impact of miR-671-5p on the relative luciferase levels as detected by a dual fluorescence reporter system. KYSE180 and EC109 cells were transfected with WT or MT luciferase reporter vector. **H.** Representative immunoblots (**left**) and plots (**right**) showing levels of FGFR2 protein in KYSE180 and EC109 cells transfected with miR-671-5p. Nc, negative control. **I.** Representative immunoblots** (left)** and plots** (right)** showing levels of FGFR2 protein in KYSE180 and EC109 cells transfected with AZD4547 or miR-671-5p.

To investigate how FGFR2 expression is regulated in ESCC, we collected six pairs of FGFR2-positive ESCC tissues and FGFR2-negative normal tissues and performed, miRNA microarray analysis (Fig. 5A). We found that the levels of twelve miRNAs were reduced, while the levels of four miRNAs were increased in tumor tissues compared with those in normal tissues (Fig. 5A, B). The reduction in levels of 12 miRNAs was further confirmed by qPCR (Fig. 5C). Next, to predict the target genes of these miRNAs, multiple databases including miRnada, RNA22, miRwalk, miRDB, and TargetScan were used. We found that miR-671-5p was the only potential miRNA that specifically targets the 3′-UTR of FGFR2 (Fig. 5D, E). We further performed bivariate correlation analysis of 35 ESCC tissues as described above, and found a significantly negative correlation between the levels of FGFR2 protein and miR-671-5p (Fig. 5F). We treated KYSE180 and EC109 cells were treated with either the wild-type or mutant luciferase reporter of miR-671-5p (Fig. 5E) as well as with negative or specific mimics and inhibitors, as described earlier. We found that transfection with miR-671-5p mimics suppressed the levels of FGFR2, while transfection with miR-671-5p inhibitor enhanced its levels in KYSE180 cells treated with wild-type vector. Furthermore with miR-671-5p mimics suppressed the levels of FGFR2, but treatment with miR-671-5p inhibitor did not affect its levels in EC109 cells treated with wild-type vector (Fig. 5G). KYSE180 cells treated with miR-671-5p mimics displayed reduced the levels of FGFR2 protein, while EC109 cells treated with miR-671-5p inhibitor showed increased levels of this protein (Fig. 5H). At the same time, we used AZD4547 as a control to confirm that miR-671 was targeting the FGFR2-3'-UTR furtherly (Fig. 5I). Therefore, our result showed that miR-671-5p suppresses the expression of FGFR2.

### miR-671-5p suppresses proliferation, colony formation, migration, invasion, and tumorigenesis of ESCC cells

**Figure 6:**
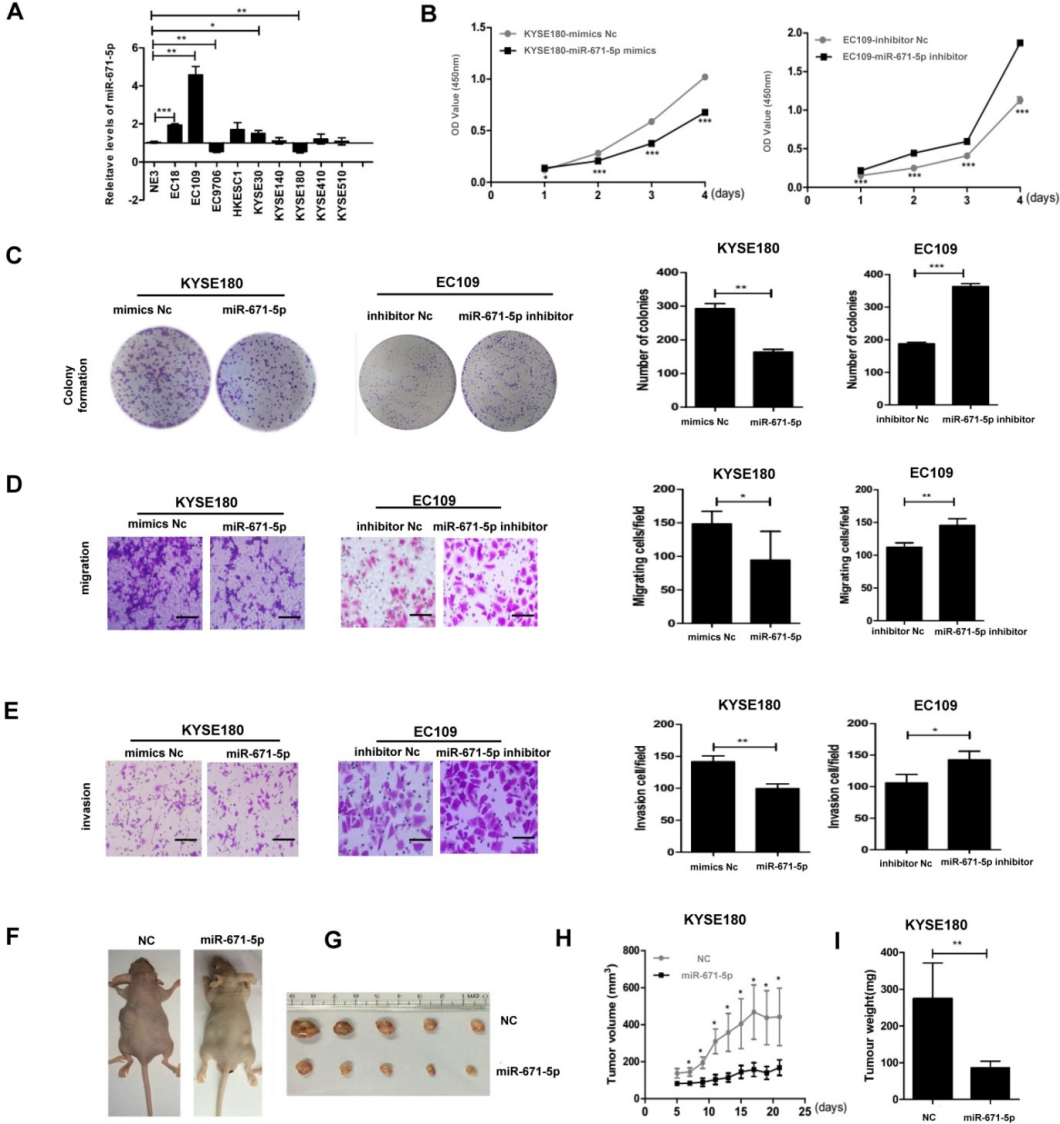
** miR-671-5p suppresses the progression of ESCC*.* A.** A plot showing the relative levels of miR-671-5p as detected by qPCR in normal esophageal cell NE3 and several ESCC cell lines. **B.** Plots showing the relative densities of KYSE180 cells treated with negative control (Nc) or miR-671-5p mimics, and EC109 cells treated with Nc or miR-671-5p inhibitor. **C-E.** Representative images and plots showing the relative levels of colony formation (**C**), migration (**D**) and invasion (**E**) in the same cells as described in panel Bar=100μm **B**. **F.** Images showing the impact of miRNAs on the development of xenograft tumors originated from KYSE180 cells in mice. **G** Images showing the solid tumor tissues collected from ESCC xenograft mice. **H,I**. A plot showing the volumes (**H**) and weights of tumors collected from mice (**I**).

We screened the levels of miR-671-5p in the same panel of ESCC cell lines as described in Figure. 1B and found that EC109 cells with the lowest levels of FGFR2 displayed the highest levels of miR-671-5p, while KYSE180 cells with the highest levels of FGFR2 exhibited the lowest levels of miR-671-5p (Fig. 6A). The rates of cell proliferation, colony formation, migration, and invasion were reduced in KYSE180 cells treated with miR-671-5p mimics, but increased in EC109 cells treated with miR-671-5p inhibitor (Fig. 6B-E). Mice injected with KYSE180 cells developed tumors of a size of approximately 1 cm in diameters and then injected with miR-671-5p mimics or negative control mimics daily for 7 days. Treatment with miR-671-5p mimics significantly suppressed the development of tumors compared to treatment with mimics Nc (Fig. 6F-I). Therefore, our findings suggested that miR-671-5p suppresses the rates of proliferation, colony formation, migration, invasion, and tumorigenesis of ESCC cells specifically through regulating the expression of FGFR2.

### miR-671-5p suppresses ERK and AKT signaling pathway through the phosphorylation of FGFR2

**Figure 7:**
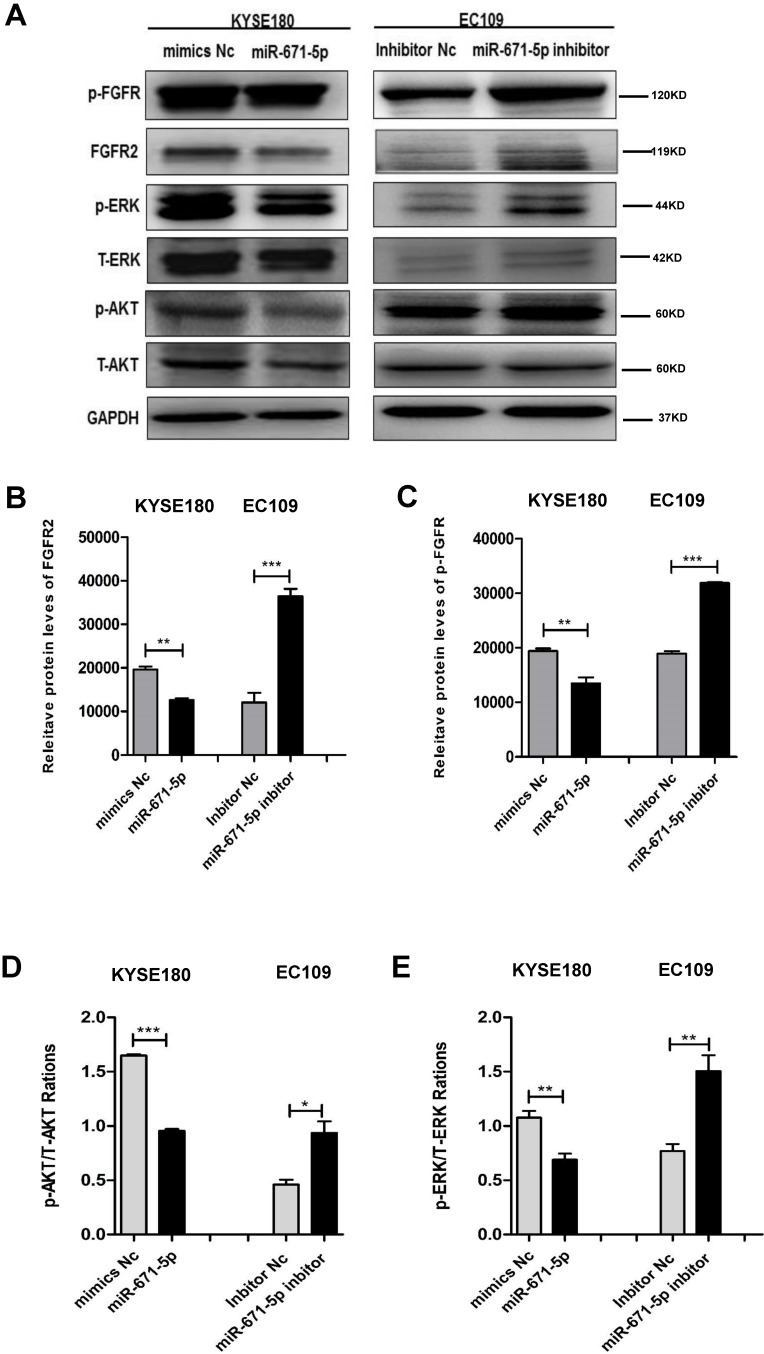
** miR-671-5p inhibits the phosphorylation of FGFR2 and signals of MAPK-ERK and PI3K-AKT pathways in ESCC cells**. **A-E.** Representative immunoblots (**A**), and plots of relative levels of FGFR2 (**B**) and p-FGFR proteins (**C**), and ratios of p-AKT to AKT (**D**) and ratios of p-ERK to ERK (**E**) in cells as described in **Fig. 6B**.

To further decipher the mechanism by which miR-671-5p suppresses the tumorigenesis of ESCC cells, we tested the effect of miR-671-5p on the expression of FGFR2 and its downstream targets. KYSE180 cells treated with miR-671-5p mimics displayed reduced levels, while EC109 cells treated with miR-671-5p inhibitor showed increased levels of total FGFR2 (Fig. 7A, B), phosphorylated FGFR (Fig. 7A, C), phosphorylated AKT (Fig. 7A, D) and phosphorylated ERK (Fig. 7A, E). Therefore, miR-671-5p suppressed the progression of ESCC by suppressing the expression of FGFR2 via inhibiting the MAPK-ERK and PI3K-AKT pathways.

## Discussion

Due to the shortage of precise therapeutic targets for ESCC, surgery is still the main line of treatment, although surgery alone has poor locoregional control and poor long-term outcome. Surgical treatment can achieve a five-year survival rate of approximately 10%-40% for patients with non-metastatic ESCC 33. However, chemoradiotherapy is the most widely accepted non-surgical therapy, but is highly toxic and occasionally lethal to patients with ESCC 34. Thus, targeted treatment is expected to have a greater potential for ESCC therapy 34.

The intracellular proteins SOX2 35, AKT1 36 and STAT3 37 have been considered as potential therapeutic targets for ESCC, but are still not used for clinical application. The FGFR variant was reported to be involved in the development of various cancers 38. High levels of FGFR2 have been associated with gastric cancer 39, 40, oral squamous cell carcinoma 41 and breast cancer 42, 43, and promote the proliferation, invasion, and metastases of tumor cells. We detected a similar trend. High levels of FGFR2 were detected in ESCC tissues, which promoted the migration, invasion, and proliferation of ESCC cells. Therefore, suppressing the expression of FGFR2 may be a potential strategy for ESCC therapy.

There are various methods to suppress the expression of FGFR2. Currently, the reported targeting molecules include inhibitors 44, 45, RNA aptamer 46-49, peptide mimetic 50, human antibody, siRNAs and synthetic miRNAs targeting the compounds of FGFRs 51-53 26. Drugs targeting FGFRs such as dovitinib, AZD4547, lucitanib, BGJ398, and JNJ-42756493 are currently in clinical trials, and may become a new line of treatment for patients with ESCC 54. In addition, miRNAs also play important roles in various types of cancers 55, 56. It has been showed that miR-494 53, miR-381-3p 41 and miR-125b 57 directly target FGFR2 to regulate cancer progression. Here we reported that miR-671-5p specifically binds with the 3′-UTR of FGFR2 to suppress the expression of FGFR2, which inhibited the proliferation, migration, and invasion of ESCC cells. Since more and more evidences indicate that miRNAs may enhance chemotherapy efficacy for multiple human cancers 58, 59, miR-671-5p may potentially be utilized as a drug in combination with other treatments for ESCC therapy in the future.

**Figure 8:**
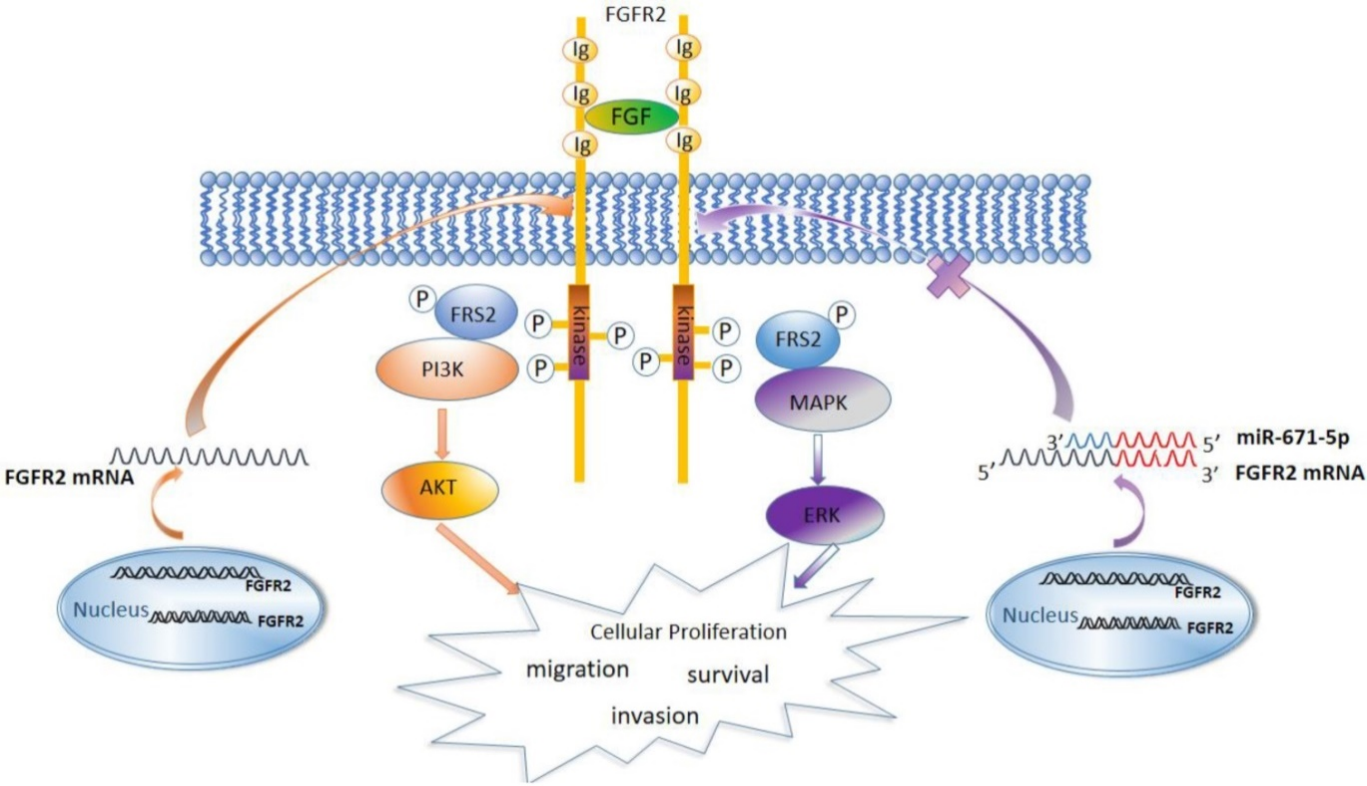
A diagram showing the mechanism by which miR671-5p suppresses ESCC progression through FGFR2.

Previous studies have showed that the FGFR family members activate the MAPK, AKT, and PLCγ signaling pathways 27. We found that FGFR2 similarly activated the MAPK-ERK and PI3K-AKT signaling pathways and increased the expression levels of the cell cycle-regulatory proteins cyclin D1 and cyclin B1. It has been reported that both cyclin D1 and cyclin B1 accelerate the cell cycle and are associated with malignance and proliferation of tumor cells 60-63. Therefore, miR-671-5p suppresses the expression of FGFR2, inhibits the MAPK-ERK and PI3K-AKT signaling pathways to block the cell cycle, and inhibits the proliferation, invasion and migration of ESCC cells and progression of ESCC (Fig. 8).

## Figures and Tables

**Table 1:** Primers used in this study

Primers	Sequences ( From 5' to 3' )
miR-5585-RT	CTCAACTGGTGTCGTGGAGTCGGCAATTCAGTTGAG ACCTGT
miR-5585-F	ACACTCCAGCTGGG CTGAATAGCTGGGACT
miR-4534-RT	CTCAACTGGTGTCGTGGAGTCGGCAATTCAGTTGAG AGACCC
miR-4534-F	ACACTCCAGCTGGG GGATGGAGGAG
miR-5703-RT	CTCAACTGGTGTCGTGGAGTCGGCAATTCAGTTGAG ACCTTC
miR-5703-F	ACACTCCAGCTGGG AGGAGAAGTCG
miR-6086-RT	CTCAACTGGTGTCGTGGAGTCGGCAATTCAGTTGAG CTCTGC
miR-6086-F	ACACTCCAGCTGGG GGAGGTTGGGAAGG
miR-4485-RT	CTCAACTGGTGTCGTGGAGTCGGCAATTCAGTTGAG TTAGGG
miR-4485-F	ACACTCCAGCTGGG TAACGGCCGCGGTA
miR-3141-RT	CTCAACTGGTGTCGTGGAGTCGGCAATTCAGTTGAG TCCTCC
miR-3141-F	ACACTCCAGCTGGG GAGGGCGGGTGGA
miR-1973-RT	CTCAACTGGTGTCGTGGAGTCGGCAATTCAGTTGAG TATGCT
miR-1973-F	ACACTCCAGCTGGG ACCGTGCAAAGGT
miR-3132-RT	CTCAACTGGTGTCGTGGAGTCGGCAATTCAGTTGAG TCCTCT
miR-3132-F	ACACTCCAGCTGGG TGGGTAGAGAAGGAGCTC
miR-671-5p-RT	CTCAACTGGTGTCGTGGAGTCGGCAATTCAGTTGAG CTCCAG
miR-671-5p-F	ACACTCCAGCTGGG AGGAAGCCCTGGAGGGG
U6-R	AACGCTTCACGAATTTGCGT
U6-F	CTCGCTTCGGCAGCACA
FGFR2-3'-UTR-R	TTGCGGCCGCGTCTTGTTAACATTAATATC
FGFR2-3'-UTR-F	CCGCTCGAGTCTTCAGGAGATGATTCTGT
FGFR2-M8-R	GCGTCTCCAACGCCAAAGAGTCTTTCGTATATTATCAAAAT
FGFR2-M8-F	CAGTGAATTTTGATAATATACGAAAGACTCTTTGGCGTTG
GAPDH-R	AAGTGGTCGTTGAGGGCAATG
GAPDH-F	CTGGGCTACACTGAGCACC
FGFR2-R	ACACTGCCGTTTATGTGTGGA
FGFR2-F	AGCCAACCTCTCGAACAGTAT

**Table 2:** Relationship between FGFR2 expression and clinical pathological parameter of ESCC and NE.

Characteristics	Total number	FGFR2 Expression level		*P*-Value	
		ESCC	NE	ESCC	NE
Age				0.99	0.63
<60	15	4.93±3.91	2.80±1.70		
>60	20	4.95±2.80	2.65±1.87		
Gender				0.62	0.03*
Female	6	4.33±2.50	1.51±1.51		
Male	29	5.07±3.43	3.00±1.67		
Pathology grade				0.98	0.33
I+II	14	4.92±3.38	3.07±1.85		
II-III+III	21	4.95±3.27	2.48±1.66		
Lymph node metastasis				0.66	0.59
Negative	13	4.62±3.31	2.92±1.97		
Positive	22	5.14±3.31	2.59±1.62		

## Abbreviations

ESCC: Esophageal Squamous Cell Carcinoma
ERK: extracellular regulated protein kinases
AKT: Protein kinase B
MAPK: mitogen-activated protein kinase
PI3K: Phosphoinositide 3-kinase
miRNA: MicroRNA
qPCR: Real-time Quantitative PCR Detecting System
STAT3: Signal transducer and activator of transcription 3
mTOR: mammalian target of rapamycin
p-AKT: phosphor-AKT
p-ERK: phosphor-ERK
